# A phase I study evaluating the pharmacokinetics, safety and tolerability of an antibody-based tissue factor antagonist in subjects with acute lung injury or acute respiratory distress syndrome

**DOI:** 10.1186/1471-2466-12-5

**Published:** 2012-02-16

**Authors:** Peter E Morris, Jay S Steingrub, Bee Y Huang, Shamay Tang, Patrick M Liu, Peter R Rhode, Hing C Wong

**Affiliations:** 1 Section on Pulmonary, Critical Care, Allergy and Immunologic Diseases, Wake Forest University School of Medicine, Winston Salem, NC, USA; 2 Division of Pulmonary and Critical Care Medicine, Baystate Medical Center, Springfield, MA, USA; 3 Altor BioScience Corp., Miramar, FL, USA; 4 Development Sciences, Genentech, Inc., South San Francisco, CA, USA

**Keywords:** Tissue Factor, Acute Lung Injury, Acute Respiratory Distress Syndrome, Clinical Trial, Phase I

## Abstract

**Background:**

The tissue factor (TF)-dependent extrinsic pathway has been suggested to be a central mechanism by which the coagulation cascade is locally activated in the lungs of patients with acute lung injury and acute respiratory distress syndrome (ALI/ARDS) and thus represents an attractive target for therapeutic intervention. This study was designed to determine the pharmacokinetic and safety profiles of ALT-836, an anti-TF antibody, in patients with ALI/ARDS.

**Methods:**

This was a prospective, randomized, placebo-controlled, dose-escalation Phase I clinical trial in adult patients who had suspected or proven infection, were receiving mechanical ventilation and had ALI/ARDS (PaO_2_/FiO_2 _≤ 300 mm). Eighteen patients (6 per cohort) were randomized in a 5:1 ratio to receive ALT-836 or placebo, and were treated within 48 hours after meeting screening criteria. Cohorts of patients were administered a single intravenously dose of 0.06, 0.08 or 0.1 mg/kg ALT-836 or placebo. Blood samples were taken for pharmacokinetic and immunogenicity measurements. Safety was assessed by adverse events, vital signs, ECGs, laboratory, coagulation and pulmonary function parameters.

**Results:**

Pharmacokinetic analysis showed a dose dependent exposure to ALT-836 across the infusion range of 0.06 to 0.1 mg/kg. No anti-ALT-836 antibody response was observed in the study population during the trial. No major bleeding episodes were reported in the ALT-836 treated patients. The most frequent adverse events were anemia, observed in both placebo and ALT-836 treated patients, and ALT-836 dose dependent, self-resolved hematuria, which suggested 0.08 mg/kg as an acceptable dose level of ALT-836 in this patient population.

**Conclusions:**

Overall, this study showed that ALT-836 could be safely administered to patients with sepsis-induced ALI/ARDS.

**Trial registration:**

ClinicalTrials.gov: NCT01438853

## Background

Acute lung injury (ALI) and acute respiratory distress syndrome (ARDS) are major causes of acute respiratory failure in patients of all ages, resulting in high rates of morbidity and mortality despite decades of clinical research. ALI/ARDS is characterized by diffuse alveolar damage leading to disruption of the alveolar capillary barrier, pulmonary edema and neutrophilic inflammation. Extravascular intra-alveolar thrombin formation and fibrin deposition, often evident as hyaline membranes lining the denuded alveolar surface, have long been recognized as pathological hallmarks of ALI/ARDS. These findings suggest that the coagulation cascade and the fibrinolytic pathway, responsible for fibrin clot clearance, are altered in patients with ALI/ARDS [1-4].

The tissue factor (TF)-dependent extrinsic pathway has been suggested as a central mechanism by which the coagulation cascade is locally activated in the lungs of patients with ALI/ARDS. TF is a transmembrane glycoprotein normally expressed on subendothelial cells in the vascular adventitia layer that is not in contact with the circulating blood [5]. Vessel injury or pathological conditions leading to the exposure TF in the vascular adventitia layer or induction of TF expression on endothelial cells and monocytes permits interactions between TF and coagulation factor VIIa (FVIIa) resulting in the formation of the high affinity TF-FVIIa complex. This complex then binds factor × (FX), converting it to the activated form FXa, which ultimately leads to thrombin formation and fibrin deposition [6]. TF-FVIIa complexes also play a role in cell signaling events mediated by the TF cytoplasmic domain and by activation of the protease activated receptors (PARs) either directing or via downstream TF-dependent coagulation proteases [1,7,8]. These signaling events stimulate proinflammatory cytokines, growth factors and chemokines, some of which further upregulate TF expression.

A direct role of TF in promoting ALI/ARDS has been suggested based on elevated levels of TF observed in plasma and pulmonary fluid of ALI/ARDS patients compared to control subjects [9-11]. These higher plasma TF levels correlated with the presence of disseminated intravascular coagulation and sepsis in patients with ALI/ARDS, and were associated with prolonged use for mechanical ventilation and increased mortality. Immunohistochemistry of the lung tissue from patients with ALI/ARDS showed prominent TF expression by alveolar epithelial cells as well as intra-alveolar macrophages and hyaline membranes [9], suggesting an active role of intra-alveolar TF in fibrin deposition within the lungs of these patients.

Consequently, development and evaluation of TF antagonists has been of interest as a therapeutic strategy for treating ALI/ARDS [1-3]. ALT-836 is a recombinant IgG4κ chimeric antibody that binds to human TF or the TF-FVIIa complex preventing the association and activation of FX, thereby inhibiting thrombin generation [12]. The results of a preclinical study in a baboon model of established *E. coli*-induced sepsis demonstrated that ALT-836, administered after the onset of the disease, was able to reverse the course of sepsis-induced lung and organ injury by reducing abnormalities in gas exchange, pulmonary hypertension, lung compliance and other clinically relevant parameters [13]. Additionally, ALT-836 has been administered to subjects with stable coronary artery disease (CAD) where it exhibited dose-dependent anticoagulant effects [14]. Based on these preclinical and clinical findings, we hypothesized that ALT-836 could serve as a potential therapeutic agent for the treatment of sepsis-induced ALI/ARDS. In this randomized, placebo-controlled, dose-escalation Phase I clinical trial, we evaluated the safety and pharmacokinetics of ALT-836 in critically ill patients with sepsis-induced ALI/ARDS.

## Methods

### Patient selection

In this randomized, placebo-controlled, assessor-blinded, dose-escalation Phase I clinical trial (ClinicalTrials.gov: NCT01438853), seventeen ICUs (13 United States, 4 Canada) received institutional review board approval to conduct the study and eight of these sites enrolled at least one patient. Study enrollment occurred between December 2004 and July 2006. Informed consent was received by each patient or his/her legal surrogate prior to enrollment. Eligible patients were at least 18 years of age, had suspected or proven infection, were receiving mechanical ventilation (≤ 48 h) and had ALI/ARDS. Diagnostic criteria for ALI/ARDS included all of the following: acute bilateral pulmonary infiltrates on a chest x-ray consistent with the presence of pulmonary edema, a ratio of arterial oxygen tension to fraction of inspired oxygen (PaO_2_/FiO_2_) of 100 to 300 mm Hg for ALI/ARDS, and the clinical absence of left atrial hypertension. The maximum period of sepsis induced ALI/ARDS prior to study drug infusion was 48 hours. Exclusion criteria included the following: platelet count < 50,000/mm^3^, prolonged prothrombin time (PT) (INR > 3) or activated partial thromboplastin time (aPTT) (> 2 × ULN), significant potential for disseminated intravascular coagulation or increased risk of bleeding (2 or more measurements of prolonged aPTT (> 1.5 × ULN), fibrinogen level < LLN, presence of petechiae, ecchymoses, or other evidence of coagulopathy) (see Additional file 1: Table A1 for complete inclusion/exclusion criteria).

### Intervention

Based on the safety profile of ALT-836 observed in a dose-escalation Phase I study in patients with stable CAD [14], this clinical trial was originally designed with five dosing cohorts (0.06, 0.1, 0.2, 0.3, and 0.4 mg/kg) of ALT-836 with six subjects in each cohort. Patients were to be randomized in a 5:1 ratio to receive ALT-836 or placebo, respectively, administered as a single intravenous bolus infusion over 15 minutes. Cohorts were enrolled sequentially. Patients were then followed for a 28-day period post study drug administration. Following the observation of adverse events (hematuria) in the 0.1 mg/kg dose group, the Data Safety Monitoring Board (DSMB) recommended including an additional 5:1 randomized dose group of 0.08 mg/kg ALT-836 or placebo administered through an intravenous infusion over 2 hours (50 cc/hr). The slower infusion rate was employed to determine if there is a relationship between maximum serum concentration of ALT-836 (C_max_) and occurrence of adverse events. The protocol was amended and the final study treatment groups included ALT-836 dosed at 0.06 (5 patients), 0.08 (5 patients), and 0.10 mg/kg (5 patients) and placebo (3 patients).

### Patient assessments

Blood samples were collected prior to administration of the study drug and subsequently post drug administration at 15 and 30 min, 1, 4, 6 (8 hr in 0.08 mg/ml cohort), 12 and 24 hrs, daily thereafter through day 7 and weekly through day 28 for measurement of coagulation, hematology and serum chemistry parameters and serum levels of ALT-836. Serum samples for detection of anti-ALT-836 antibodies were obtained pre-dose and at 2 and 4 weeks post dosing. Safety assessments performed during this study included investigator reported adverse events, routine laboratory parameters including blood gas measurements and chest x-rays, vital signs measurements, physical examinations (baseline and 1, 7, and 28 days post dosing) and electrocardiograms. Additional assessments included evaluating surrogate parameters for resource utilization (*i.e*. ICU length of stay, ventilation free days, etc.).

### Assay methods

Plasma samples for quantification of coagulation parameters were stored at -70°C until assayed at a central laboratory. Standard PT assays using recombinant human TF (RecombiPlasTin; Beckman Coulter, Miami, FL) or rabbit brain thromboplastin (PT-Fibrinogen HS; Beckman Coulter) and standard aPTT assays were conducted to assess the activity of plasma coagulation factors. PT-derived fibrinogen assays were performed using RecombiPlasTin reagent. Thrombin generation was evaluated based on levels of prothrombin fragment 1 + 2 (F_1+2_) and D-dimer assessed by a commercially available enzyme linked immunosorbent assay (ELISA) (Dade Behring, Deerfield, IL) and an antibody-coated latex agglutination assay (Beckman Coulter), respectively.

Concentration of ALT-836 in serum samples was determined using a validated sandwich ELISA, as described previously [14]. Serum titer of anti-ALT-836 antibodies of different isotypes, if present, was determined by a validated bridging ELISA method employing plate-bound ALT-836 as a capture reagent and HRP-labeled ALT-836 as a detection reagent.

### Study endpoints and stopping rules

The endpoints of this study were to evaluate the safety and pharmacokinetics of ALT-836 in patients with suspected or proven sepsis-induced ALI/ARDS. Major bleeding was defined as any of the following occurring within 2 weeks of infusion of ALT-836: intracranial hemorrhage; clinically overt or occult bleeding requiring surgical intervention; or transfusion of four or more units of packed red blood cells over a 48-hour period. Minor bleeding was defined as clinically overt or occult bleeding that did not meet the definition of major bleeding. Predefined stopping rules included early study termination pending DSMB review/recommendation if major bleeding occurred in two patients infused with ALT-836 within a cohort and study termination or dose modification if one ALT-836 treated patient experienced a major bleeding event.

### Statistical analysis

All randomized patients received study drug or placebo as planned and analysis was conducted on an intent-to-treat basis. Safety, pharmacokinetic and other data were grouped by study treatment and dose level. Safety variables were tabulated and presented for all patients who received ALT-836 or placebo. Non-compartmental methods (WinNonlin 5.0, Pharsight Corp., Mountain View, CA) were used to determine the pharmacokinetic parameters. Continuous variables were expressed as mean ± SD and were compared using Student's *t *test or the Wilcoxon rank sum test, where appropriate. Testing for an association between the dose of ALT-836 and bleeding was performed using a *χ*^2 ^test for trend. Analysis of the variance was used to assess changes in variables from baseline following study drug administration. A two-sided *P *value of less than 0.05 was considered statistically significant. All *P *values are unadjusted for multiple testing.

## Results

Baseline characteristics of the 18 patients enrolled in this Phase I clinical trial are shown in Table 1. Of these patients, one patient of the 0.08 mg/kg ALT-836 cohort died (acute hypoxia, unrelated to study drug) during the study, with the remaining patients completing the 28-day follow-up period.

**Table 1:** Baseline characteristics

Characteristics	Placebo (n = 3)	0.06 mg/kg ALT-836 (n = 5)	0.08 mg/kg ALT-836 (n = 5)	0.10 mg/kg ALT-836 (n = 5)
Age, yr	49.7 ± 24.3	40.4 ± 18.2	54.0 ± 16.9	54.6 ± 13.7
Male, n (%)	1 (33.3)	1 (20.0)	2 (40.0)	0 (0.0)
Baseline APACHE II Score	22.7 ± 14.2	20.2 ± 5.5	20.0 ± 3.1	19.2 ± 5.1
Baseline respiratory variables				
PO_2_/FIO_2_	226 ± 51	205 ± 65	221 ± 64	200 ± 48
Respiratory rate, bpm	28.7 ± 4.2	20.3 ± 6.9	20.60 ± 3.3^a^	19.8 ± 7.2
pH	7.34 ± 0.09	7.35 ± 0.07	7.44 ± 0.04	7.39 ± 0.06
PEEP, cm H_2_O	10.0 ± 5.0	10.3 ± 5.6	10.4 ± 6.2	9.2 ± 4.1
Baseline coagulation & hematology variables				
Prothrombin time, sec	17.0 ± 2.4	12.1 ± 1.5	14.8 ± 1.9	13.9 ± 0.3
aPTT, sec	32.9 ± 0.3	29.1 ± 2.5	31.1 ± 1.8	29.2 ± 1.7
Platelets, 10^6^/ml	238 ± 115	256 ± 105	357 ± 309	244 ± 43
Hemoglobin, g/dl	10.7 ± 0.3	9.5 ± 1.2	10.0 ± 1.4	10.0 ± 1.8

APACHE II, Acute Physiology and Chronic Health Evaluation; PEEP, Positive end-expiratory pressure; aPTT, activated partial thromboplastin time

Values are means ± SD unless otherwise noted

^a ^*P *< 0.05 compared to placebo group

### Pharmacokinetics and immunogenicity

Study drug-treated patients of the first two cohorts received 0.06 mg/kg or 0.1 mg/kg ALT-836 as a single bolus intravenous infusion over 15 minutes, whereas the third cohort received 0.08 mg/kg ALT-836 through a 2 hr infusion at 50 cc/hr. Pharmacokinetic analysis showed a dose dependent exposure to ALT-836 across the infusion range of 0.06 to 0.1 mg/kg (Table 2). Thus, increasing the duration of infusion to 2 hrs for the 0.08 mg/kg dose group did not decrease C_max _or overall exposure levels (AUC_INF_) as these values remained linear with dose. The terminal elimination half-life for ALT-836 was equivalent across dose levels with mean values ranging from 18.5 to 22.6 hrs. Volumes of distribution were about 50 ml/kg, consistent with plasma volume in the vasculature.

**Table 2:** Pharmacokinetic parameters

	0.06 mg/kg ALT-836	0.08 mg/kg ALT-836	0.1 mg/kg ALT-836
t_1/2 _term., hour	18.5 ± 5.9	22.6 ± 3.1	22.6 ± 6.2
C_max_, ng/ml (nM)	1,300 ± 560 (8.6)	1,490 ± 340 (9.9)	1,800 ± 250 (12.0)
Vd, ml/kg	46.1 ± 13.1	47.7 ± 16.0	54.5 ± 6.6
Cl, ml/hr/kg	1.9 ± 1.0	1.5 ± 0.7	1.8 ± 0.4
AUC_INF_, hr·ng/ml	39,000 ± 24,200	59,900 ± 20,500	59,900 ± 16,600

t_1/2 _term., terminal half-life; Cmax, maximum serum concentration; Vd, volume of distribution; Cl, total body clearance: AUC_INF_, area under the drug concentration versus time curve extrapolated to infinity

Values are means ± SD

There was no detectable serum anti-ALT-836 antibody activity in any of the ALT-836-treated patients (n = 15).

### Adverse events

Four patients encountered non-fatal serious adverse events (SAEs) during the study, including bilateral pulmonary embolism (0.06 mg/kg ALT-836-treated patient), hypoxic respiratory failure (0.1 mg/kg ALT-836-treated patient), worsening acute renal failure (placebo-treated patient), and worsening anemia and empyema (placebo-treated patient).

Of the 18 subjects enrolled in this trial, 16 subjects reported a total of 87 adverse events (AEs) during the study. Of these, 35 (40%) were mild in intensity, 48 (55%) were moderate in intensity, and 4 (5%) AEs were severe in intensity. The investigators attributed 16 of these AEs as "related to study treatment" (2 in placebo group, 3 in 0.06 mg/kg ALT-836; 6 in 0.08 mg/kg ALT-836, and 5 in 0.1 mg/kg ALT-836 groups). A summary of the incidence of treatment emergent AEs is presented Table 3 and a detailed listing of the AEs is provided in Additional file 2: Table A2.

**Table 3:** Adverse events by treatment group

	Placebo	0.06 mg/kg ALT-836	0.08 mg/kg ALT-836	0.10 mg/kg ALT-836	Total
	(n = 3)	(n = 5)	(n = 5)	(n = 5)	(n = 18)
Mortality by study day 28 (treatment related)	0 (0)	0 (0)	1 (0)	0 (0)	1 (0)
Patients with non-fatal SAEs (treatment related)	2 (1^a^)	1 (0)	0 (0)	1 (1^b^)	5 (2)
Total number of AEs (treatment related)	20 (2)	18 (3)	29 (5)	20 (6)	87 (16)
Patients with hematuria AEs (treatment related)	0 (0)	2 (2)	2 (2)	5 (4)	9 (8)
Patients with anemia AEs (treatment related)	2 (2)	1 (1)	2 (2)	3 (0)	8 (5)

^a ^Worsening anemia and empyema reported as possible related to placebo treatment

^b ^Hypoxic respiratory failure (study day 23) secondary to hospital-acquired pneumonia reported as possible related to study drug treatment

The most frequent AE reported in patients treated with ALT-836 was hematuria or worsening hematuria (n = 9) (Table 3). These AEs were rated as mild to moderate and assessed by the clinical investigator as possibly or probably related to study drug in 8 of the 9 patients. On average, hematuria started 6.7 hours after study drug administration (range 3 - 19 hr), and resolved by 29.1 hours (range 6-53 hrs) post-onset without the need of medical intervention. No patient with hematuria became hemodynamically unstable and there was no decrease in platelet counts, prolongation of PT and aPTT time or increase in serum D-dimer and F_1+2 _levels. There was also no significant difference in markers of renal function (BUN, creatinine) between patients with or without hematuria and no signs of capillary fragility (petechiae, ecchymoses, conjunctival bleeding) were observed during the duration of hematuria. Besides hematuria, no major bleeding or other spontaneous minor bleeding events were observed in any patient receiving ALT-836.

Anemia was reported for 6 patients receiving ALT-836 as well as in two of three patients receiving placebo (Table 3). These adverse events were assessed as study drug related in 3 of 6 ALT-836-treated patients and in both of the placebo treated patients, one of which (as noted above) experienced anemia as a serious adverse event.

Aside from these events, there were no other adverse events or serious adverse events judged to be related to ALT-836 treatment by the clinical investigators.

### Assessment of clinical laboratory measurements and patient outcomes

There was no significant treatment effect on PT, platelet counts, aPTT, or plasma levels of D-dimer, prothrombin fragment F1 + 2 or fibrinogen at any ALT-836 dose. At the dose levels administered, the ALT-836 concentration observed in the patients' plasma were previously shown to be insufficient to prolong PT driven by the large excess of TF reagent added in the standard PT assay, though inhibition of clot formation and thrombin generation was observed in a whole blood assay [14]. Additionally, treatment with high levels of a different TF antagonist reportedly did not alter levels of either coagulation factors or markers of thrombin generation in patients with ALI/ARDS [15]. Thus, these laboratory parameters of coagulation may not be useful in assessing the pharmacodynamic effects of TF-targeted therapies.

As part of the safety assessment, evaluation of oxygenation index and patient outcomes was conducted. There was no significant treatment effect at any dose of ALT-836 on PO_2_/FIO_2 _ratio while patients were on mechanical ventilation. Assessment of hospital resource indices also did not reveal significant differences in ventilation free days or duration of hospital stay following ALT-836 treatment, though patients receiving 0.1 mg/kg ALT-836 showed an improvement in ICU-free days at day 28 when compared to placebo treated patients (mean ± SD: 8.7 ± 8.1 (placebo) vs. 20.0 ± 2.4 (ALT-836), *P *= 0.036) (Table 4). Although this result is encouraging, it should be noted that this study was not sufficiently powered to assess ALT-836 efficacy and that because of the large inherent variation in outcome parameters observed in this patient population, preliminary evaluation of efficacy end points will likely require a placebo-controlled Phase II trial with at least 45 subjects per treatment arm [16]. The overall mortality rate observed in this study was lower than that reported in recently published studies in ALI/ARDS subjects [15]. However, this also likely reflects the small number of subjects in this study since ongoing follow-up studies with larger numbers of the same subject population verified that placebo-treated subjects have a mortality rate consistent with historic values (H. Wong, personnel communication).

**Table 4:** Hospital indices by treatment group

	Placebo	0.06 mg/kg ALT-836	0.08 mg/kg ALT-836	0.1 mg/kg ALT-836
Days on ventilator	25.5 ± 25.7	14.1 ± 23.1	15.5 ± 10.4	6.4 ± 2.6
Days in ICU	29.2 ± 26.5	15.2 ± 22.1	16.4 ± 10.1	7.1 ± 2.4
Days in hospital	33.3 ± 29.1	29.9 ± 45.6	25.4 ± 12.6	16.7 ± 10.5
ICU free days at SD28^a^	8.7 ± 8.1	17.4 ± 9.8	8.4 ± 9.0	20.0 ± 2.4^c^
Ventilator free days at SD28^b^	11.0 ± 9.8	18.8 ± 10.6	8.4 ± 11.2	21.0 ± 3.1

Values are means ± SD

^a ^Mean number of days to study day 28 (SD28) that the patients were not admitted to the ICU. Patients that did not survive to SD28 were assigned zero ICU free days

^b ^Mean number of days to SD28 that the subjects were achieved unassisted breathing. Patients that did not survive to SD28 were assigned zero ventilator free days

^c ^*P *< 0.05 compared to placebo group

## Discussion

In a number of pre-clinical models, blockade of TF is capable of preventing mortality during systemic infection and inflammation, inhibiting endothelial cell injury induced by anoxia-reoxygenation and permeability of endothelial cells during systemic inflammation, reducing lung and renal fibrin deposition, and limiting proliferation, migration and invasion of malignant cells [13,17,18]. In humans, TF levels are elevated in plasma and pulmonary fluid of ALI/ARDS patients compared to control subjects [9-11]. Taken together, these findings suggest that TF may represent an important therapeutic target for treatment of systemic inflammatory diseases such as ALI/ARDS [1,2]. However, bleeding-related complications resulting from therapeutic inhibition of TF activity remain a major concern. This was recently illustrated in a Phase II clinical trial in ALI/ARDS patients where administration of the TF-FVIIa complex inhibitor, active site inactivated FVIIa (FVIIai), resulted in a dose-depended increase in major bleeding events (3.8% for the low dose to 17% for the highest dose) and an increased 28-day mortality rate [15]. In contrast to the FVIIai study, we found that ALT-836, an antibody-based TF antagonist, was well tolerated by ALI/ARDS patients with no drug-related deaths or major bleeding event observed following treatment with dose levels from 0.06 to 0.1 mg/kg. A similar safety profile (*i.e*. no serious bleeding or drug-related SAEs) for ALT-836 at a single dose levels up to 0.3 mg/kg was observed in 26 patients with CAD who were also receiving daily anti-platelet aspirin therapy [14]. In the trial reported here, there was an ALT-836 dose-dependent increase in mild to moderate hematuria observed in some patients that typically started 6 to 8 hours after study drug administration and spontaneously resolved approximately 24 hours post onset. These minor bleeding episodes did not result in hemodynamic instability and were not associated with alterations in renal function or coagulation parameters. Interestingly, analysis of the pooled data of ALT-836-treated patients did not show a correlation between hematuria and higher ALT-836 serum levels or exposure. Thus, it is possible that other clinical confounders, including baseline/previous history of hematuria or concurrent use of urinary catheters, influenced the development of hematuria following ALT-836 administration.

The dose levels of ALT-836 administered, while relatively low in comparison to those of other therapeutic antibodies, appear to be within the range required to inhibit disease-associated TF activity. In the current study, the results of pharmacokinetic analysis indicate that ALT-836 administered at 0.06 mg/kg to 0.1 mg/kg provided a maximum serum concentration of 9 to 12 nM, respectively. *In vitro *studies have demonstrated that these ALT-836 concentrations are sufficient to inhibit > 90% of TF cofactor activity at a broad range of TF concentrations (0.005-2.3 nM) [12]. It is noteworthy that disease-associated, elevated TF levels found in undiluted pulmonary edema fluid of patients with ALI/ARDS ranged from 0.5 and 2 nM, approximately 100-fold higher than plasma TF levels of the same patients [9]. Similarly higher levels of TF-dependent procoagulant activity in bronchoalveolar lavage (BAL) fluid of ARDS patients was observed three days after clinical recognition of ARDS and decreased thereafter [11]. Thus one would expect that ALT-836 at dose levels tested in the present study when administered shortly after diagnosis of ALI/ARDS should provide sufficient antibody to block pulmonary and intravascular TF activity for several days. Although ALT-836 levels were not measured in patient's pulmonary fluid of patients, we have previously shown that the antibody could be readily detected at the expected nanomolar level in BAL fluid collected from baboons in a sepsis-induced organ failure model [13], suggesting that ALT-836 is capable of distributing between the vascular and alveolar compartments. The pharmacokinetic analysis also revealed differences in the antibody half-life between patients with ALI/ARDS and CAD. Serum ALT-836 levels typically displayed exponential decay kinetics with a terminal half-life of about 21 hours in ALI/ARDS patients, whereas a biphasic decay with an initial plateau phase and a median 72 hour terminal half life was observed in patients with stable CAD [14]. While patient management may have affected the antibody pharmacokinetics, it is possible that elevated levels of the TF antigen in the vascular and pulmonary compartments of the ALI/ARDS patients contributed to the observed increase rate of serum ALT-836 clearance. Such antigen-antibody interactions have been reported to play a role in the elimination kinetics of a variety of other therapeutic antibodies [19].

The abundant evidence of extensive cross-talk between the coagulation and the inflammatory systems have led to numerous but unsuccessful studies utilizing anti-thrombotic and fibrinolytic agents for treatment of ALI/ARDS and sepsis [15,16,20-24]. Although most of these agents are classified as anti-coagulants, they are known to have other physiological functions in addition to their anti-thrombotic activities. For example, activated protein C (APC) not only regulates the coagulation pathway by inactivating factor VIIIa and factor Va which decreases thrombin mediated inflammation, but also binds to the endothelial cell protein C receptor to inhibit endotoxin-induced intracellular calcium fluxes and NF-κB nuclear translocation. These effects influence gene expression profiles, inhibit leukocyte chemotaxis and adhesion to activated endothelium, and inhibit endothelial cell apoptosis [25-29]. By stimulating cell signaling, APC can modulate the cellular response to infectious insults leading to anti-inflammatory, cytoprotective and barrier-protective properties which may contribute significantly to APC effectiveness for severe sepsis treatment [30]. TF, either alone or in complex with FVIIa, has also been shown to exhibit many physiological effects in systemic inflammation, which differ from those of many anti-coagulants including APC. Besides its role in triggering the coagulation pathway, the TF-FVIIa complex per se activates PAR2, which plays a crucial role in inflammatory responses, fibrosis and tissue remodeling [31,32]. In a mouse model, activation of PAR2 in the lung resulted in airway constriction, pulmonary edema and increased lung vascular and epithelial permeability [33]. Lung PAR2 activation also induced acidosis, hypoxemia, hypercapnia and increase leukocyte infiltration, suggesting that PAR2 activation can trigger a variety of inflammatory responses associated with lung injury [33]. Similarly, recent studies provide evidence that TF/FVIIa-mediated FXa activation in the lung drives fibrotic responses via a PAR1-dependent signaling mechanism [34,35]. Consequently, the effectiveness of anti-coagulants as anti-inflammatory agents may be largely dependent on the relevance of particular pro-inflammatory signaling processes at the systemic and local levels under different pathological conditions. Thus, the enthusiasm for employing novel anti-coagulants as therapeutic approaches for ALI/ARDS should not be tempered by the lack of clinical effectiveness of a particular anti-coagulant in the treatment of these inflammation-associated debilitating diseases.

## Conclusions

Identification of an increased incidence of hematuria at the 0.10 mg/kg dose level supported an acceptable safe dose of ALT-801 at 0.08 mg/kg in patients with sepsis-induced ALI/ARDS in this Phase I trial. Pharmacokinetic analysis indicated that ALT-836 administrated at 0.06 to 0.1 mg/kg could provide sufficient serum antibody levels to inhibit elevated TF activity reported in ALI/ARDS patients. Taken together, these results suggest that evaluation of the clinical activity of this antibody in patients with ALI/ARDS is warranted. Thus, a follow-up randomized, double-blind, placebo-controlled trial with a target enrollment of 150 patients is currently being conducted to further assess the safety and efficacy of single and multiple dose ALT-836 treatment in patients with sepsis-induced ALI/ARDS (ClinicalTrials.gov ID: NCT00879606).

## Abbreviations

aPTT: Activated partial thromboplastin time; APC: activated protein C; FVIIai: active site inactivated FVIIa; ALI: acute lung injury; APACHE: Acute Physiology and Chronic Health Evaluation; ARDS: acute respiratory distress syndrome; AEs: adverse events; AUC_INF_: area under the drug concentration versus time curve extrapolated to infinity; BAL: bronchoalveolar lavage; CAD: coronary artery disease; DSMB: Data Safety Monitoring Board; ELISA: enzyme linked immunosorbent assay; FVIIa: factor VIIa; FX: factor X; C_max_: maximum serum concentration; PEEP: positive end-expiratory pressure; PAR: protease activated receptors; F_1+2_: prothrombin fragment 1 + 2; PT: prothrombin time; PaO_2_/FiO_2_: ratio of arterial oxygen tension to fraction of inspired oxygen; SAEs: serious adverse events; t_1/2 _term: terminal half-life; TF: tissue factor; Cl: total body clearance; Vd: volume of distribution.

## Competing interests

At the time of this study, BYH, ST, PRR, and HCW were employees and equity holders of Altor BioScience Corp.

## Authors' contributions

PEM and JSS were study investigators and contributed equally to this work; PEM, JSS, BYH and HCW made contributions to the conception and/or design of the study; PEM, JSS, BYH, ST and HCW contributed to initiation and conduct of the study; PML and PRR contributed to data analysis; PEM, JSS, PML, PRR and HCW contributed to the interpretation of the study data; PEM, JSS, PRR and HCW contributed to the development and drafting of the manuscript. All authors read and approved the manuscript prior to submission.

## Pre-publication history

The pre-publication history for this paper can be accessed here:

http://www.biomedcentral.com/1471-2466/12/5/prepub

## Supplementary Material

Additional file 1 **Table A1. Criteria for enrollment and treatment**. Click here for file

Additional file 2 **Table A2. Summary of treatment-emergent adverse events**. Click here for file
