# Supplementary material for: A phase I study evaluating the pharmacokinetics, safety and tolerability of an antibody-based tissue factor antagonist in subjects with acute lung injury or acute respiratory distress syndrome
Source: BMC Pulm Med. 2012 Feb 16;12:5. doi: 10.1186/1471-2466-12-5 (PMC3299584; doi:10.1186/1471-2466-12-5)
Supplement: Additional file 1 — Table A1. Criteria for enrollment and treatment. [file 1471-2466-12-5-S1.PDF]

Additional file 1

Table A1: Criteria for enrollment and treatment

Inclusion Criteria
**1.** 18 years of age or older; **2.** Suspected or proven bacterial infection. Subjects with suspected bacterial infection must have had evidence of an infection such as the presence of white blood cells in a normally sterile body fluid (except blood), perforated viscus, chest x-ray consistent with pneumonia and associated with purulent sputum production, or a clinical syndrome associated with a high probability of bacterial infection (e.g., ascending cholangitis); **3.** Receiving positive pressure ventilation through an endotracheal tube; **4.** Have ALI/ARDS, defined as having all of the following: a) chest x-ray performed after intubation showing bilateral infiltrates consistent with pulmonary edema (patchy, diffused, homogeneous, or asymmetric), b) hypoxemia: ratio of partial arterial oxygen pressure (PaO_2) to FiO_2 (PaO_2/FiO_2) taken after intubation, that is ≤300 mm Hg and >100 mm Hg, and c) no clinical evidence of left atrial hypertension. If measured, pulmonary arterial occlusion pressure ≤18 mmHg; and**5.** Provide signed informed consent, authorization in accordance with the Health Insurance Portability Accountability Act of 1996 (HIPAA) and agree to comply with all protocol-specified procedures and follow-up evaluations.
**Exclusion Criteria**
**1.** Mechanically or chemically-induced ALI/ARDS (including burns, trauma, and near drowning); **2.** End-stage lung disease; **3.** Decompensated congestive heart failure, **4.** Authorization to withdraw life support, **5.** Hemoglobin persistently <8.0 g/dL; **6.** Subjects who have any one of the following: a) platelet count <50,000/mm3, b) prolonged prothrombin time (PT), defined as international normalized ratio (INR) >3, c) prolonged activated partial thromboplastin time (aPTT), defined as >2 times the upper limit of normal, d) in the opinion of the investigator, having significant potential for disseminated intravascular coagulation (DIC); **7.** Subjects who have two or more of the following: a) prolonged aPTT, defined as >1.5 times the upper limit of normal, b) fibrinogen level below the lower limit of normal, and c) presence of petichiae, ecchymoses, or other evidence of coagulopathy; **8.** Subjects who have a history of one or more of the following: a) hematuria (microscopic or gross), b) urinary tract neoplasia, c) nephrolithiasis, d) glomerulonephritis, and e) active urinary tract infection (UTI); **9.** Bleeding disorders (including gross hematuria and gastrointestinal bleeding requiring medical intervention unless definitive surgery has been performed) within the past 6 weeks or vasculitis with diffused alveolar hemorrhage; **10.** Diagnosis of bleeding peptic ulcer disease within the previous 2 months; **11.** Congenital bleeding diatheses such as hemophilia; **12.** Treatment with anti-platelet, anti-coagulant agents, or non-steroidal anti-inflammatory drugs (NSAIDs) as described below or anticipated requirement for such therapy within 72 hours following infusion of study drug. a) therapeutic heparin: unfractionated heparin to treat an active thrombotic or embolic event within eight hours prior to study drug infusion, or low molecular weight heparins used at therapeutic dose within the 12 hours prior to study drug infusion; b) prophylactic heparin: unfractionated heparin >15,000 units/day, or low molecular weight heparins greater than the recommended dose in the product label for prophylactic use; c) warfarin, if used within 7 days prior to study drug infusion and if prothrombin time is prolonged beyond the upper limit of normal for the institution; d) thrombolytic treatment within 3 days prior to study drug infusion (e.g., streptokinase, tPA, rPA, and urokinase); e) glycoprotein IIb/IIIa antagonists within 7 days prior to study drug infusion, f) aspirin or any aspirin containing compound within 3 days prior to study drug infusion, and g) APC infusion within 72 hours prior to study drug infusion; **13.** Major trauma (within previous 6 months) or trauma subjects at an increased risk of bleeding (e.g., flail chest, significant contusion to lung, liver, or spleen, retroperitoneal bleed, pelvic fracture, or compartment syndrome); **14.** History of severe head trauma that required hospitalization, intracranial surgery, or stroke within previous 3 months or any history of intracerebral arteriovenous malformation, cerebral aneurysm, or central nervous system mass lesion. Subjects with an epidural catheter or who anticipate receiving an epidural catheter during study drug infusion; **15.** Major surgery within the previous 3 days, any postoperative subject with evidence of active bleeding, or any subject with planned or anticipated surgery within 72 hours after study drug infusion. History of abnormal bleeding during surgical procedures; **16**. Chronic renal failure, defined as a calculated glomerular filtration rate (GFR) ≤20 mL/min; **17.** Subjects with baseline aspartate transaminase (AST) or alanine transaminase (ALT) level >5 times the upper limit of normal. Subjects with known esophageal varices, chronic jaundice, biopsy proven cirrhosis, or chronic ascites; **18.** History of organ transplant (including bone marrow); **19.** Subjects with malignancy having a life expectancy <6 months; **20.** Known human immunodeficiency virus (HIV) positive with $CD4^+$ T Cell count <200/uL; **21.** Women who are pregnant or nursing; **22.** Participation in another clinical research study within 30 days before administration of study drug, with the exception of participation in studies involving noninvasive monitoring medical devices; **23.** Any prior treatment with a murine or chimeric antibody; **24.** Subjects who are moribund and where death is perceived to be imminent (within 72 hours after screening); **25**. Subjects who have persistent hypotension (systolic blood pressure <90 mm Hg or mean arterial blood pressure <60 mm Hg) not responding to fluid or vasopressor administration; subjects who require more than two vasopressors; norepinephrine at doses >0.1 mcg/kg/min, epinephrine >0.1mcg/kg/min, dopamine >15 mcg/kg/min, or phenylephrine >333 mcg/minute to maintain mean arterial pressure (MAP) ≥60 mm Hg after adequate fluid resuscitation; and **26.** Any medical condition which in the opinion of the investigator would interfere with optimal participation in the study or that would produce a significant risk to a subject.
**Treatment**
Subjects should be administered ALT-836 or placebo within 48 hours of meeting the screening criteria
