# Supplementary material for: A phase I study evaluating the pharmacokinetics, safety and tolerability of an antibody-based tissue factor antagonist in subjects with acute lung injury or acute respiratory distress syndrome
Source: BMC Pulm Med. 2012 Feb 16;12:5. doi: 10.1186/1471-2466-12-5 (PMC3299584; doi:10.1186/1471-2466-12-5)
Supplement: Additional file 2 — Table A2. Summary of treatment-emergent adverse events. [file 1471-2466-12-5-S2.PDF]

## Additional file 2

**Table A2:** Summary of treatment-emergent adverse events

| Adverse Event (MedDRA Preferred Term) | Number of Subjects(Treatment-Related[a]) Placebo | 0.06 mg/kg ALT-836 | 0.1 mg/kg ALT-836 | 0.08 mg/kg ALT-836 | Total |
|---|---|---|---|---|---|
| ***Blood and Lymphatic System Disorders*** | | | | | |
| Anemia | 2 (2) | 1 (1) | 1 (0) | 2 (1) | 6 (4) |
| Lymphocytic infiltration | 1 (0) | 0 (0) | 0 (0) | 0 (0) | 1 (0) |
| ***Cardiac Disorders*** | | | | | |
| Atrial fibrillation | 1 (0) | 0 (0) | 0 (0) | 0 (0) | 1 (0) |
| Bradycardia | 0 (0) | 0 (0) | 0 (0) | 2 (0) | 2 (0) |
| Tachycardia | 0 (0) | 1 (0) | 0 (0) | 0 (0) | 1 (0) |
| ***Congenital, Familial, and Genetic Disorders*** | | | | | |
| Macroglossia | 0 (0) | 0 (0) | 0 (0) | 1 (0) | 1 (0) |
| ***Gastrointestinal Disorders*** | | | | | |
| Diarrhea | 0 (0) | 1 (0) | 0 (0) | 0 (0) | 1 (0) |
| Nausea | 0 (0) | 1 (0) | 1 (0) | 0 (0) | 2 (0) |
| Pancreatitis | 1 (0) | 0 (0) | 0 (0) | 0 (0) | 1 (0) |
| ***General Disorders and Administrative Site Conditions*** | | | | | |
| Bloody discharge | 0 (0) | 0 (0) | 1 (1) | 0 (0) | 1 (1) |
| Edema peripheral | 1 (0) | 0 (0) | 0 (0) | 0 (0) | 1 (0) |
| Pyrexia | 0 (0) | 0 (0) | 1 (0) | 0 (0) | 1 (0) |
| ***Hepatobiliary Disorders*** | | | | | |
| Cholecystitis | 0 (0) | 1 (0) | 0 (0) | 0 (0) | 1 (0) |
| ***Infections and Infestations*** | | | | | |
| Bacteremia | 0 (0) | 1 (0) | 0 (0) | 0 (0) | 1 (0) |
| Candiduria | 0 (0) | 1 (0) | 0 (0) | 0 (0) | 1 (0) |
| Empyema | 1 (0) | 0 (0) | 0 (0) | 0 (0) | 1 (0) |
| Oral candidiasis | 1 (0) | 0 (0) | 0 (0) | 0 (0) | 1 (0) |
| Pneumonia | 1 (0) | 0 (0) | 1 (0) | 1 (0) | 2 (0) |
| Sepsis | 1 (0) | 0 (0) | 0 (0) | 0 (0) | 1 (0) |
| Tinea cruris | 0 (0) | 0 (0) | 0 (0) | 1 (0) | 1 (0) |
| Urinary tract infection | 0 (0) | 1 (0) | 0 (0) | 0 (0) | 1 (0) |
| Vulvovaginal mycotic infection | 0 (0) | 0 (0) | 1 (0) | 0 (0) | 1 (0) |
| ***Injury, Poisoning, and Procedural Complications*** | | | | | |
| Procedural pain | 0 (0) | 1 (0) | 0 (0) | 0 (0) | 1 (0) |
| Weaning failure | 0 (0) | 0 (0) | 0 (0) | 1 (0) | 1 (0) |
| ***Investigations*** | | | | | |
| Blood creatinine increased | 0 (0) | 0 (0) | 0 (0) | 1 (0) | 1 (0) |
| Blood urea increased | 0 (0) | 0 (0) | 0 (0) | 1 (0) | 1 (0) |
| Hematocrit decreased | 0 (0) | 0 (0) | 1 (0) | 0 (0) | 1 (0) |
| Hemoglobin decreased | 0 (0) | 0 (0) | 1 (0) | 1 (1) | 2 (1) |
| Oxygen saturation decreased | 0 (0) | 0 (0) | 0 (0) | 1 (0) | 1 (0) |
| ***Metabolism and Nutrition Disorders*** | | | | | |
| Hyperkalemia | 0 (0) | 0 (0) | 1 (0) | 0 (0) | 1 (0) |
| Hypernatremia | 1 (0) | 0 (0) | 0 (0) | 1 (0) | 2 (0) |
| Hypoglycemia | 0 (0) | 1 (0) | 0 (0) | 0 (0) | 1 (0) |
| Hypokalemia | 0 (0) | 0 (0) | 3 (0) | 1 (0) | 4 (0) |
| Hypomagnesemia | 0 (0) | 0 (0) | 1 (0) | 0 (0) | 1 (0) |
| Hypophosphatemia | 1 (0) | 0 (0) | 0 (0) | 0 (0) | 1 (0) |
| ***Nervous System Disorders*** | | | | | |
| Hemiparesis | 1 (0) | 0 (0) | 0 (0) | 0 (0) | 1 (0) |
| ***Psychiatric Disorders*** | | | | | |
| Agitation | 0 (0) | 1 (0) | 0 (0) | 2 (0) | 3 (0) |
| Anxiety | 0 (0) | 0 (0) | 0 (0) | 1 (0) | 1 (0) |
| Delirium | 1 (0) | 0 (0) | 0 (0) | 1 (0) | 2 (0) |
| Hallucination | 0 (0) | 0 (0) | 0 (0) | 1 (0) | 1 (0) |
| Insomnia | 0 (0) | 0 (0) | 1 (0) | 0 (0) | 1 (0) |
| ***Renal and Urinary Disorders*** | | | | | |
| Hematuria | 0 (0) | 2 (2) | 5 (4) | 2 (1) | 9 (7) |
| Renal failure acute | 1 (0) | 0 (0) | 0 (0) | 0 (0) | 1 (0) |
| ***Respiratory, Thoracic, and Mediastinal Disorders*** | | | | | |
| Asthma | 0 (0) | 1 (0) | 0 (0) | 0 (0) | 1 (0) |
| Bronchospasm | 1 (0) | 0 (0) | 0 (0) | 0 (0) | 1 (0) |
| Hypoxia | 0 (0) | 0 (0) | 1 (1) | 2 (0) | 3 (1) |
| Pleural effusion | 1 (0) | 0 (0) | 0 (0) | 0 (0) | 1 (0) |
| Pulmonary edema | 1 (0) | 0 (0) | 0 (0) | 0 (0) | 1 (0) |
| Pulmonary embolism | 0 (0) | 1 (0) | 0 (0) | 0 (0) | 1 (0) |
| Tachypnea | 0 (0) | 0 (0) | 0 (0) | 1 (0) | 1 (0) |
| ***Skin and Subcutaneous Tissue Disorders*** | | | | | |
| Rash | 1 (0) | 1 (0) | 0 (0) | 1 (0) | 3 (0) |
| ***Vascular Disorders*** | | | | | |
| Hypertension | 0 (0) | 1 (0) | 0 (0) | 0 (0) | 1 (0) |
| Hypotension | 0 (0) | 1 (0) | 0 (0) | 1 (0) | 2 (0) |
| **Total Number of AEs** | **20 (2)** | **18 (3)** | **20 (6)** | **29 (5)** | **87 (16)** |
| **Total Number of Subjects with AEs** | **3 (2)** | **4 (2)** | **5 (4)** | **4 (2)** | **16 (10)** |

[a] Treatment-Related AEs had a possible, probable, or definite relationship to study medication in the opinion of the Investigator.
